# The interaction between cooling and hypoxia on the rate of peripheral and central fatigue development of the knee extensors

**DOI:** 10.1186/2046-7648-4-S1-A42

**Published:** 2015-09-14

**Authors:** Alex Lloyd, Simon Hodder, Margherita Raccuglia, Yifen Qiu, George Havenith

**Affiliations:** 1 Environmental Ergonomics Research Centre, Design School, Loughborough University, Loughborough, UK

## Introduction

High altitude often comprises hypobaric hypoxia and cold ambient temperatures. However, research examining human performance during these stressors in combination is sparse [1]. Previous findings have reported that the rate of fatigue additively increases when hypoxia and cold are combined [2]. However this study investigated small muscle groups (forearm flexors) using a fixed duration (closed) exercise protocol. Thus, the present study sought to examine whether volitional exhaustion or task failure (during an open protocol) of the larger knee extensor muscles would result in a similar additive effect during combined hypoxic-cold exposure.

## Methods

Nine physically active males were exposed to four conditions in a balanced order. The conditions were control/normoxic thermoneutrality, hypoxic thermoneutrality, normoxic cold and hypoxic cold. Thermoneutral conditions were 23°C and cold conditions were 5°C. Hypoxic exposures were 13% oxygen (~4000 m). Subjects were dressed in shorts and socks. After a 40 minute rest period, participants carried out dynamic knee extension at a fixed intensity (35[6] W) until failure. After every 110 seconds of exercise, participants performed an isometric maximal voluntary contraction (iMVC; 2 second) with twitch interpolation to quantify voluntary and peripheral fatigue. To test data at each time point for significance, a two-way (2 × 2) repeated measures ANOVA was used.

## Results

Rectal temperature was unaffected by condition (p > 0.3). Muscle (3 depth mean) and skin (7 point mean) temperature decreased by 3.8°C (0.4) and 5.4°C (0.1) in cold conditions, compared to 0.8°C (0.3) and 0.3°C (0.1) in neutral conditions (effect of temperature = p < 0.001). There was no effect of hypoxia on body temperature (effect of hypoxia = p > 0.2). Peripheral arterial oxygen saturation was significantly reduced to 85% (1) in hypoxia compared to 99% (1) in normoxia (effect of hypoxia = p < 0.001). In response to exercise, independent exposure to hypoxia and cold reduced time to task failure by 505 (105) seconds (p = 0.002) and 190 (73) seconds (p = 0.006) respectively, compared to 915 (122) seconds in control. During combined hypoxic cold, exercise time was reduced further (589 (110) seconds compared to control); however there was no significant interaction between stressors (p=0.198). The absolute reduction in time to task failure was not additive (e.g. 695-seconds); however the relative influence of hypoxia and cold were similar in the presence of the other stressor (-48% (6) and -51% (5) for hypoxia; -21% (7) and -20% (3) for cold), supporting an independent effect. The rate of increase in peripheral fatigue was also faster (p < 0.005) during independent exposure to hypoxia and cold compared to control (4.9%.min^-1 ^ (0.9) and 1.6%.min^-1 ^ (0.8) respectively). The combined effect of hypoxic-cold on peripheral fatigue rate was additive (7.6%.min^-1^ (1.1)) with no interaction (p = 0.525). Volitional (central) fatigue was unaffected by time (p = 0.327) or condition (p > 0.15) in this study.

## Conclusion

The results indicate that when compared to exercise in thermoneutral or normoxic conditions, both cold and hypoxia induce significant reductions in time to task failure. Additionally, the time to failure is decreased further when these stressors are combined, and their relative influence is not interactive. The data suggest that the rate of peripheral fatigue development is the primary factor behind the additive effects of hypoxic-cold exposure on fatigue and task failure.
